# Attenuation of Metabolic Syndrome by EPA/DHA Ethyl Esters in Testosterone-Deficient Obese Rats

**DOI:** 10.3390/md16060182

**Published:** 2018-05-24

**Authors:** Nikhil S. Bhandarkar, Senthil Arun Kumar, Jarad Martin, Lindsay Brown, Sunil K. Panchal

**Affiliations:** 1 School of Health and Wellbeing, Faculty of Health, Engineering and Sciences, University of Southern Queensland, Toowoomba QLD 4350, Australia; Nikhil.Bhandarkar@usq.edu.au (N.S.B.); senthil.ibt@gmail.com (S.A.K.); Lindsay.Brown@usq.edu.au (L.B.); 2 Functional Foods Research Group, Institute for Agriculture and the Environment, University of Southern Queensland, Toowoomba QLD 4350, Australia; 3 Department of Radiation Oncology, Calvary Mater Newcastle, Waratah NSW 2298, Australia; Jarad.Martin@calvarymater.org.au; 4 Genesis Cancer Care, Lake Macquarie Private Hospital, Gateshead NSW 2290, Australia

**Keywords:** testosterone, obesity, metabolic syndrome, leuprolide, EPA/DHA ethyl esters

## Abstract

Inducing testosterone deficiency, as the standard treatment of prostate cancer, may cause metabolic disorders including insulin resistance, dyslipidemia, central obesity, cardiovascular diseases, and type 2 diabetes. This study measured responses to testosterone deficiency in high-carbohydrate, high-fat (H) diet-fed rats. We then tested whether eicosapentaenoic acid (EPA)/docosahexaenoic acid (DHA) ethyl esters (Omacor) reversed these metabolic changes. Male Wistar rats (8–9 weeks old) were divided into eight groups with four groups fed corn starch and four groups fed H diet. For each diet, one group received diet only; one group was orchidectomized; one group was given leuprolide (gonadotrophin-releasing hormone agonist, 2 mg/kg every 4th week); and the last group was treated with leuprolide and their diet was supplemented with 3% Omacor for the last eight weeks. The protocol was for 16 weeks. Leuprolide worsened metabolic syndrome symptoms and cardiovascular function, and orchidectomy produced greater responses. In H fed leuprolide-treated rats, Omacor decreased systolic blood pressure and left ventricular diastolic stiffness, reduced infiltration of inflammatory cells and collagen deposition in the heart, and reduced lipid accumulation and inflammatory cell infiltration without improving liver damage. These results suggest that Omacor has potential to attenuate metabolic complications in prostate cancer patients with induced testosterone deprivation.

## 1. Introduction

Metabolic syndrome is the constellation of insulin resistance, impaired glucose tolerance, dyslipidemia, hypertension, and obesity that increases the risk for development of cardiovascular disease and type 2 diabetes, with an increasing prevalence in the last few decades [1]. Metabolic syndrome, potentially caused by imbalances in energy intake and expenditure, increases morbidity and mortality and is one of the leading preventable causes of death [2]. Further, low testosterone concentrations have been associated with many of these complications including insulin resistance, hyperinsulinemia, dyslipidemia, and cardiovascular disorders [3,4,5,6]. Prostate cancer is the second most commonly diagnosed cancer worldwide; in men with prostate cancer, the presence of metabolic syndrome was associated with worse oncologic outcomes, in particular with more aggressive tumor features and biochemical recurrence [7,8]. The major therapies for prostate cancer rely on induction of testosterone deficiency by orchidectomy or gonadotrophin-releasing hormone (GnRH) agonists such as leuprolide [9]. However, GnRH agonists increase the risk of development of diabetes and cardiovascular disease, increase fat mass, and decrease lean mass [10,11,12].

Diet interventions targeting these metabolic syndrome parameters would be a potential approach to decrease the risk of diabetes and cardiovascular disease in prostate cancer survivors treated with induced testosterone deficiency, and possibly slow down tumor progression. The health benefits of omega-3 fatty acids such as eicosapentaenoic acid (EPA) and docosahexaenoic acid (DHA) in metabolic syndrome and cardiovascular diseases have been reviewed [13,14,15]. Omega-3 fatty acids (EPA and DHA, 1.8 g/day for 26 weeks) decreased expression of genes associated with inflammation and atherogenesis-associated pathways [16]. Omega-3 fatty acids reduced inflammatory markers and cardiovascular disease risk factors [17,18]. Mixed EPA:DHA interventions decreased inflammation and cardiovascular disease risk components with increased activities of antioxidant enzymes [19]. In a randomized controlled trial, daily doses of 300 mg EPA and 200 mg of DHA for eight weeks reduced high-sensitivity C-reactive protein, fasting blood glucose, and triglyceride concentrations in hypertensive and/or diabetic obese patients [20].

Previously, we have characterized a diet-induced rat model that mimics the changes observed in human metabolic syndrome [21]. This study used this model to test three hypotheses. Firstly, we investigated whether orchidectomy worsened metabolic syndrome in rats fed a high-carbohydrate, high-fat diet [21]. Secondly, we investigated whether rats with testosterone deficiency following 4-weekly leuprolide injections developed similar pathophysiological changes to the high-carbohydrate, high-fat diet as the rats with orchidectomy. Thirdly, we investigated whether a commercially-available mixture of ethyl esters of EPA and DHA (Omacor) reversed the cardiovascular, liver, and metabolic parameters in these leuprolide-treated high-carbohydrate, high-fat diet-fed rats. Our hypothesis was that this mixture of EPA and DHA esters has potential as a treatment for metabolic syndrome in prostate cancer patients treated long-term with androgen deprivation.

## 2. Results

### 2.1. Effects of High-Carbohydrate, High-Fat Diet

#### 2.1.1. Dietary Intakes, Body Composition, and Metabolic Parameters

**Figure 1:**
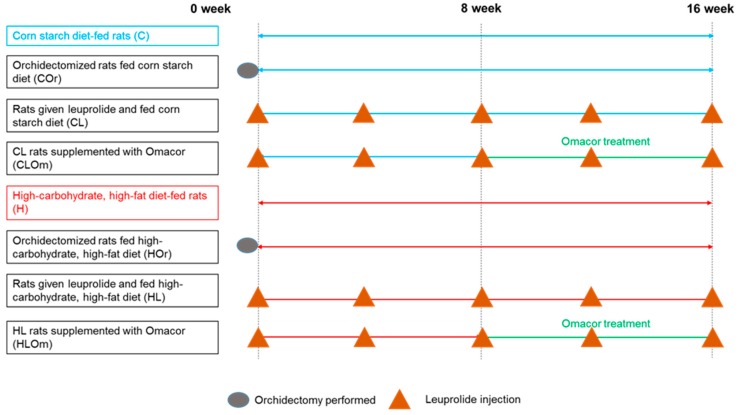
Study design to identify effects of testosterone deficiency and intervention with Omacor.

**Figure 2:**
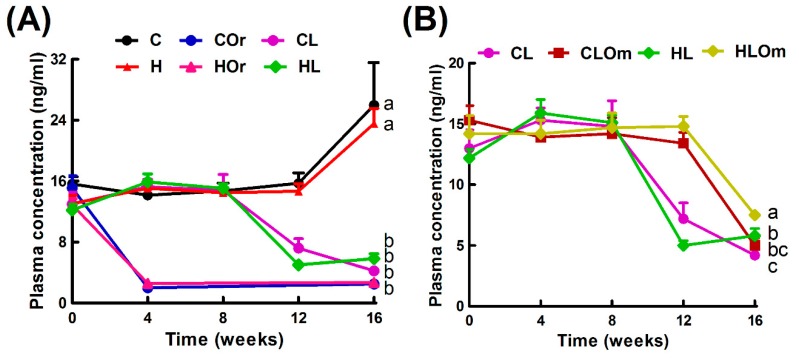
Plasma total testosterone concentrations in corn starch diet-fed rats (C), orchidectomized rats fed corn starch diet (COr), leuprolide-treated rats fed corn starch diet (CL), high-carbohydrate high-fat diet-fed rats (H), orchidectomized rats fed high-carbohydrate high-fat diet (HOr), and leuprolide-treated rats fed high-carbohydrate high-fat diet (HL) (**A**); and in CL rats, corn starch diet-fed, leuprolide and Omacor-treated rats (CLOm), HL rats, and high-carbohydrate, high-fat diet-fed, leuprolide and Omacor-treated rats (HLOm) (**B**). Data are shown as mean ± SEM, *n* = 6–10/group. End-point means without a common alphabet significantly differ, *p* < 0.05.

Figure 1 represents the experimental design for this study. High-carbohydrate, high-fat diet (H) did not alter plasma total testosterone concentrations compared to corn starch diet-fed rats (C) (Figure 2A). H rats showed increased body weight, body weight gain, feed efficiency, abdominal circumference, basal blood glucose concentrations, area under the curve for glucose, plasma concentrations of total cholesterol, triglycerides, non-esterified fatty acids (NEFA), whole-body fat mass, retroperitoneal fat, epididymal fat, and omental fat compared to C rats (Table 1). Energy intake was increased in H rats despite reductions in water intake and food intake compared to C rats while lean mass, bone mineral content, and bone mineral density were unchanged between C and H rats (Table 1).

#### 2.1.2. Cardiovascular and Liver Function

**Figure 3:**
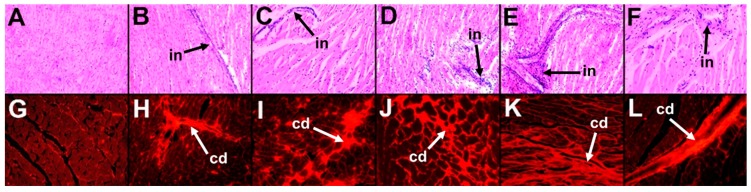
Effects of orchidectomy and leuprolide on the structure of the heart. Top row represents hematoxylin and eosin staining of left ventricle showing inflammatory cell infiltration (“in”, 20×) while the bottom row represents picrosirius red staining of left ventricle showing collagen deposition (“cd”, 40×) from corn starch diet-fed rats (**A**,**G**); orchidectomized rats fed corn starch diet (**B**,**H**); leuprolide-treated rats fed corn starch diet (**C**,**I**); high-carbohydrate high-fat diet-fed rats (**D**,**J**); orchidectomized rats fed high-carbohydrate high-fat diet (**E**,**K**); and leuprolide-treated rats fed high-carbohydrate high-fat diet (**F**,**L**).

**Figure 4:**
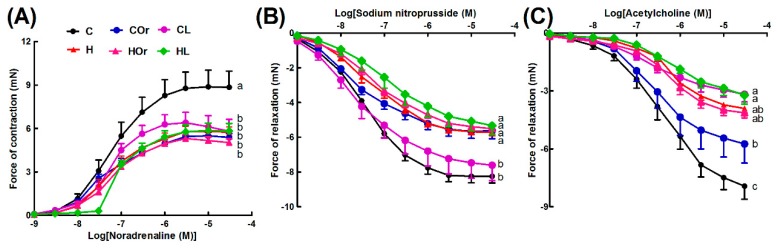
Cumulative concentration-response curves for: noradrenaline (**A**); sodium nitroprusside (**B**); and acetylcholine (**C**) in thoracic aortic rings from corn starch diet-fed rats (C), orchidectomized rats fed corn starch diet (COr), leuprolide-treated rats fed corn starch diet (CL), high-carbohydrate high-fat diet-fed rats (H), orchidectomized rats fed high-carbohydrate high-fat diet (HOr), and leuprolide-treated rats fed high-carbohydrate high-fat diet (HL). Data are shown as means ± SEM, *n* = 10–12/group. End-point means without a common alphabet significantly differ, *p* < 0.05.

H diet induced infiltration of inflammatory cells and fibrosis in hearts (Figure 3D,J) compared to C rats (Figure 3A,G). H rats showed reduced aortic responses to noradrenaline, sodium nitroprusside, and acetylcholine compared to C rats (Figure 4A–C). H diet increased heart rate, left ventricular posterior wall thickness during systole (LVPWs), cardiac output, estimated LV mass, systolic blood pressure, and left ventricular diastolic stiffness (Table 2). Other cardiovascular parameters were unchanged between C and H rats (Table 2).

**Figure 5:**
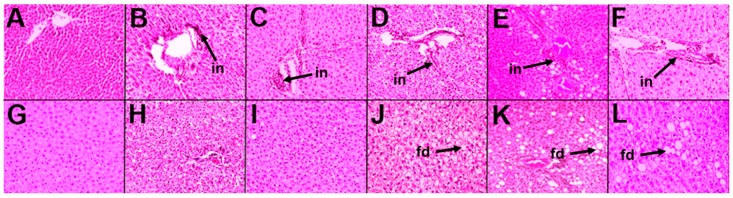
Effects of orchidectomy and leuprolide on the structure of the liver. Top row represents hematoxylin and eosin staining of liver showing inflammatory cell (“in”, 20×) while the bottom row represents hematoxylin and eosin staining of liver showing fat deposition (“fd”, 20×) from corn starch diet-fed rats (**A**,**G**); orchidectomized rats fed corn starch diet (**B**,**H**); leuprolide-treated rats fed corn starch diet (**C**,**I**); high-carbohydrate high-fat diet-fed rats (**D**,**J**); orchidectomized rats fed high-carbohydrate high-fat diet (**E**,**K**); and leuprolide-treated rats fed high-carbohydrate high-fat diet (**F**,**L**).

C diet did not induce inflammation or fat deposition in liver (Figure 5A,G), while H diet induced inflammation and fat deposition in liver (Figure 5D,J). H diet increased liver weight and plasma activities of alanine transaminase (ALT) and aspartate transaminase (AST) (Table 2).

### 2.2. Bilateral Orchidectomy

#### 2.2.1. Dietary Intakes, Body Composition, and Metabolic Parameters

Orchidectomy rapidly reduced plasma total testosterone concentrations in both corn starch diet-fed (COr) and high-carbohydrate, high-fat diet-fed (HOr) rats compared to their respective non-orchidectomized rats (C and H rats), confirming the effectiveness of orchidectomy (Figure 2A). Orchidectomy did not change body weight, feed efficiency, or intakes of food, water, and energy in COr or HOr rats compared to C or H rats, respectively (Table 1). Body weight gain was increased in COr rats compared to C rats, whereas body weight gain was unchanged between HOr and H rats (Table 1). Abdominal circumference, basal blood glucose concentrations, and area under the curve for glucose were higher in orchidectomized rats fed either C or H diet compared to their respective non-orchidectomized controls (Table 1). Plasma concentrations of total cholesterol, triglycerides, and non-esterified fatty acids (NEFA) were unchanged between the controls and their respective orchidectomized rats (Table 1). Orchidectomy reduced total lean mass in COr and HOr rats compared to C and H rats, respectively (Table 1). In contrast, the total fat mass was unchanged in C and COr rats but increased in HOr rats compared to H rats (Table 1). Bone mineral content increased in HOr rats compared to H rats but was unchanged in COr and C rats while bone mineral density was unchanged (Table 1). Retroperitoneal fat increased in COr and HOr rats compared to C and H rats, respectively, while omental fat did not change between C and COr or H and HOr rats (Table 1).

#### 2.2.2. Cardiovascular and Liver Function

Orchidectomy in COr rats increased systolic blood pressure and left ventricular diastolic stiffness while all other cardiovascular parameters were similar to C rats (Table 2). Orchidectomy induced inflammation and fibrosis in hearts of COr rats (Figure 3B,H) compared to C rats (Figure 3A,G). Orchidectomy reduced aortic responses to noradrenaline, sodium nitroprusside, and acetylcholine in COr rats compared to C rats (Figure 4A–C). HOr rats had lower heart rate and cardiac output along with higher systolic blood pressure than H rats (Table 2). Orchidectomy worsened inflammation and fibrosis induced by H diet in HOr rats (Figure 3E,K). Aortic responses to noradrenaline, sodium nitroprusside, and acetylcholine were unchanged in HOr rats compared to H rats (Figure 4A–C).

Orchidectomy induced inflammation and fat deposition in livers of COr rats (Figure 5B,H) which were absent in livers from C rats. HOr rats had inflammation and fat deposition in livers similar to H rats (Figure 5E,K). No changes in liver weight or ALT and AST activities were observed between C and COr or H and HOr rats (Table 2).

### 2.3. Leuprolide Treatment

#### 2.3.1. Dietary Intakes, Body Composition, and Metabolic Parameters

Plasma total testosterone concentrations decreased after eight weeks from initial injection in rats treated with leuprolide and fed corn starch diet (CL) compared to C rats (Figure 2A). Leuprolide treatment did not change body weight, body weight gain, feed efficiency, or water and food intakes in CL rats compared to C rats; however, energy intakes were increased in CL rats compared to C rats after 16 weeks (Table 1). Abdominal circumference, basal blood glucose concentrations, and area under the curve for glucose were higher in CL rats compared to C rats (Table 1). Plasma concentrations of total cholesterol, triglycerides, and NEFA were unchanged between CL and C rats (Table 1). Leuprolide treatment did not change fat mass of CL rats compared to C rats while the lean mass was reduced in CL rats compared to C rats (Table 1). Bone mineral content and bone mineral density did not change between CL and C rats (Table 1). Retroperitoneal fat increased in CL rats compared to C rats, while omental and epididymal fat did not change between C and CL rats (Table 1).

Plasma testosterone concentrations decreased after eight weeks from initial injection in rats treated with leuprolide and fed high-carbohydrate, high-fat diet (HL) compared to H rats (Figure 2A). Leuprolide treatment did not change body weight, body weight gain, feed efficiency, or water and food intakes in HL rats compared to H rats (Table 1). Energy intakes were increased in HL groups compared to H rats after 16 weeks (Table 1). Abdominal circumference and area under the curve for glucose were higher in HL rats compared to H rats while basal blood glucose concentrations did not change in HL rats compared to H rats (Table 1). Plasma concentrations of triglycerides and NEFA were higher in HL rats compared to H rats while plasma concentrations of total cholesterol were not different between H and HL groups (Table 1). Leuprolide treatment had no effects on the lean or fat mass in HL rats compared to H rats. Bone mineral content decreased in HL rats compared to H rats, whereas bone mineral density did not change with leuprolide treatment (Table 1). Retroperitoneal fat increased in HL rats compared to H rats, while omental and epididymal fat did not change between H and HL rats (Table 1).

#### 2.3.2. Cardiovascular and Liver Function

CL rats had increased left ventricular internal diameter during diastole (LVIDd), left ventricular internal diameter during systole (LVIDs), diastolic and systolic volumes, and systolic wall stress along with decreased fractional shortening and ejection fraction compared to C rats (Table 2). In CL rats, leuprolide induced inflammation (Figure 3C) and cardiac fibrosis (Figure 3I) and reduced aortic responses to noradrenaline and acetylcholine while not changing the response to sodium nitroprusside in CL rats compared to C rats (Figure 4A–C). Leuprolide induced inflammation, but failed to induce fat deposition in livers of CL rats (Figure 5C,I). No changes in liver weight or ALT and AST activities were observed between C and CL rats (Table 2).

HL rats had decreased heart rate and fractional shortening with increased LVIDd, LVIDs, and systolic volume compared to H rats (Table 2). HL rats showed inflammation and fibrosis in the heart (Figure 3F,L) and had similar aortic responses as in H and HOr rats (Figure 4A–C). HL rats showed inflammation and fat deposition in liver (Figure 5F,L) and no changes in liver weight or ALT and AST activities were observed between H and HL rats (Table 2).

### 2.4. Omacor Treatment

#### 2.4.1. Dietary Intakes, Body Composition, and Metabolic Parameters

Plasma total testosterone concentrations were reduced in rats treated with leuprolide, fed corn starch diet and supplemented with Omacor (CLOm) from eight weeks after initial injection, as in CL rats, and there were no differences in plasma total testosterone concentrations between CL and CLOm rats at 16 weeks (Figure 2B). Omacor treatment in CLOm rats starting eight weeks after the initial leuprolide injection decreased body weight and body weight gain even after increasing food and energy intakes (Table 3). Omacor reduced the feed efficiency, abdominal circumference, basal blood glucose concentrations, and area under the curve in CLOm rats (Table 3). Plasma concentrations of total cholesterol and NEFA were decreased while triglycerides did not change when compared to CL rats (Table 3). Total body lean mass, total body fat mass, retroperitoneal fat, and epididymal fat were decreased in CLOm rats compared to CL rats (Table 3).

Plasma total testosterone concentrations were reduced in rats treated with leuprolide, fed high-carbohydrate, high-fat diet and supplemented with Omacor (HLOm) from eight weeks after initial injection, as in HL rats, and there were no differences in plasma total testosterone concentrations between HL and HLOm rats at 16 weeks (Figure 2B). Omacor treatment did not change body weight and feed efficiency but it increased water intake (fructose-containing water) in HLOm rats compared to HL rats (Table 3). Food and energy intakes did not change in HLOm rats compared to HL rats after 16 weeks (Table 3). Body weight gain was decreased in HLOm rats compared to HL rats during the intervention period (Table 3). Abdominal circumference did not change in HLOm rats compared to HL rats (Table 3). The area under the curve for glucose load was higher in HLOm rats compared to HL rats while basal blood glucose concentrations were decreased in HLOm rats compared to HL rats (Table 3). Plasma concentrations of total cholesterol, triglycerides, and NEFA were reduced in HLOm rats compared to HL rats (Table 3). In contrast to the plasma lipid profile, Omacor decreased lean mass in HLOm rats compared to HL rats while it increased fat mass in HLOm rats compared to HL rats (Table 3). Bone mineral content was unchanged while bone mineral density increased in HLOm rats. Retroperitoneal fat and epididymal fat did not change in HL and HLOm rats while omental fat was increased in HLOm rats in comparison to HL rats (Table 3).

#### 2.4.2. Cardiovascular and Liver Function

**Figure 6:**
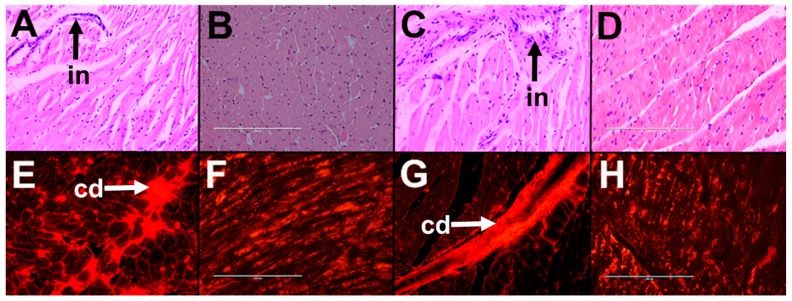
Effects of Omacor on leuprolide-induced changes in the structure of the heart. Top row represents hematoxylin and eosin staining of left ventricle showing inflammatory cell infiltration (“in”, 20×) while the bottom row represents picrosirius red staining of left ventricle showing collagen deposition (“cd”, 40×) from leuprolide-treated rats fed corn starch diet (**A**,**E**); leuprolide-treated rats fed corn starch diet supplemented with Omacor (**B**,**F**); leuprolide-treated rats fed high-carbohydrate high-fat diet (**C**,**G**); and leuprolide-treated rats fed high-carbohydrate, high-fat diet supplemented with Omacor (**D**,**H**).

**Figure 7:**
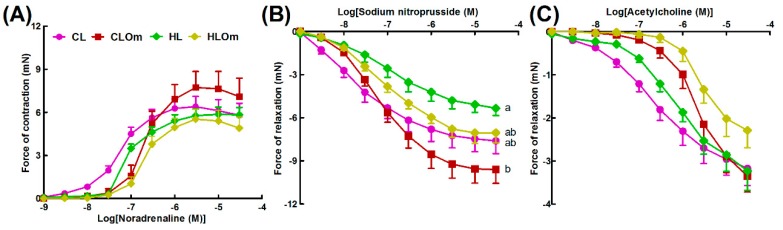
Cumulative concentration-response curves for: noradrenaline (**A**); sodium nitroprusside (**B**); and acetylcholine (**C**) in thoracic aortic rings from leuprolide-treated rats fed corn starch diet (CL), leuprolide-treated rats fed corn starch diet supplemented with Omacor (CLOm), leuprolide-treated rats fed high-carbohydrate high-fat diet (HL), and leuprolide-treated rats fed high-carbohydrate, high-fat diet supplemented with Omacor (HLOm). Data are shown as mean ± SEM, *n* = 10–12/group. End-point means without a common alphabet significantly differ, *p* < 0.05.

**Figure 8:**
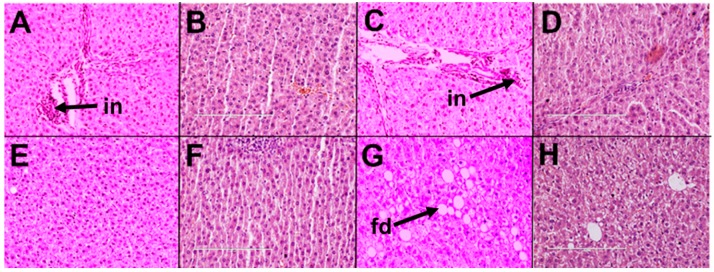
Effects of Omacor on leuprolide-induced changes in the structure of the liver. Top row represents hematoxylin and eosin staining of liver showing inflammatory cell infiltration (“in”, 20×) while the bottom row represents hematoxylin and eosin staining of liver showing fat deposition (“fd”, 20×) from leuprolide-treated rats fed corn starch diet (**A**,**E**); leuprolide-treated rats fed corn starch diet supplemented with Omacor (**B**,**F**); leuprolide-treated rats fed high-carbohydrate high-fat diet (**C**,**G**); and leuprolide-treated rats fed high-carbohydrate, high-fat diet supplemented with Omacor (**D**,**H**) rats.

In CLOm rats, blood pressure and left ventricular diastolic stiffness constant decreased with Omacor treatment compared to CL rats (Table 4). Omacor treatment reduced inflammation and fibrosis in hearts of CLOm rats (Figure 6B,F) compared to CL rats (Figure 6A,E). Omacor treatment did not improve aortic responses in CLOm rats compared to CL rats (Figure 7A–C). Liver weights decreased and ALT activity increased in CLOm rats (Table 4). Omacor treatment attenuated inflammation in livers of CLOm rats (Figure 8B) compared to CL rats (Figure 8A).

In HLOm rats, LVIDs, diastolic volume, systolic volume, systolic wall stress, estimated left ventricular mass, right ventricular weight, and left ventricular weight were increased when compared to HL rats (Table 4). LVPWs, fractional shortening, ejection fraction, systolic blood pressure, and left ventricular diastolic stiffness decreased in HLOm rats when compared with HL rats (Table 4). Omacor treatment reduced inflammation and fibrosis in hearts of HLOm rats (Figure 6D,H) compared to HL rats (Figure 6C,G). Omacor treatment did not improve aortic responses in HLOm rats compared to HL rats (Figure 7A–C). Omacor treatment attenuated inflammation and fat deposition in livers of HLOm rats (Figure 8D,H) compared to HL rats (Figure 8C,G). No changes were observed in liver wet weight or plasma ALT and AST activities between HL and HLOm rats (Table 4).

## 3. Discussion

Prostate cancer is the second most commonly diagnosed cancer worldwide with 14% of newly diagnosed cases of cancer and it is associated with about 6% of total cancer deaths [22]. The association between metabolic syndrome and prostate cancer focused on the function of insulin, IGF-1, and their receptors as strategic factors in downstream signaling pathways that stimulate tumor growth [23]. Standard treatment of prostate cancer is androgen deprivation either by orchidectomy or treatment with GnRH agonists such as leuprolide. These interventions decreased testosterone concentrations to lead to improved health status of prostate cancer patients by restricting the process of tumorigenesis in the prostate leading to tumor regression, easing of urinary symptoms and bone pain, and prolonged survival [24,25]. However, patients on androgen deprivation therapy show detrimental changes in body composition such as weight gain, loss of muscle mass, increased fat mass, and decreased muscle strength, with increased fasting glucose, triglycerides, and cholesterol concentrations [26].

In the present study using diet-induced metabolic syndrome in rats, we firstly demonstrated that orchidectomy is associated with worse pathophysiological signs of metabolic syndrome in rats fed a high-carbohydrate, high-fat diet. These metabolic changes mimic the progression present in humans with metabolic syndrome [21]. Secondly, we demonstrated that rats with testosterone deficiency induced either by leuprolide or orchidectomy developed similar pathophysiological changes when fed with high-carbohydrate, high-fat diet. Thirdly, we demonstrated that a commercially-available mixture of ethyl esters of EPA and DHA (Omacor) reversed the cardiovascular complications such as decreased blood pressure and left ventricular wall stiffness, reduced hepatic damage such as decreased inflammation and fat deposition in liver and decreased plasma lipid concentrations in these leuprolide-treated high-carbohydrate high-fat diet-fed rats. Thus, the positive changes against metabolic syndrome with the dietary supplementation have validated our hypothesis that this mixture of EPA and DHA ethyl esters has potential as a nutraceutical approach for reducing components of metabolic syndrome in prostate cancer patients treated long-term with leuprolide.

Orchidectomy and treatment with GnRH agonists such as leuprolide are successful interventions for prostate cancer by initiating testosterone deficiency [27], although orchidectomy is more effective for the initiation of glandular apoptosis and atrophy [28]. Testosterone deficiency produces complications including increased central obesity, increased triglycerides concentrations and elevated fasting plasma glucose concentrations [10,29,30,31,32]. Leuprolide, although associated with these metabolic complications, has shown tolerability in routine clinical use [33]. Thus, it is important to treat complications associated with GnRH agonist therapy to provide benefits against prostate cancer while minimizing the development of metabolic and cardiovascular complications.

This study compared the physiological changes produced by testosterone deficiency, either by orchidectomy or leuprolide treatment, and whether these changes worsen during a high-carbohydrate, high-fat diet. In this study, corn starch was used as the control diet as corn starch is a slowly digestible, low-glycemic carbohydrate [21]. Unlike fructose in high-carbohydrate, high-fat diet, corn starch in this study did not induce clinical signs of metabolic syndrome [21,34,35], although some starches based on their digestibility can induce some signs of metabolic syndrome [36]. High-carbohydrate, high-fat diet induced metabolic syndrome with obesity, dyslipidemia, hypertension and impaired glucose tolerance along with changes in liver and heart structure and function [21].

Testosterone deficiency was induced by either orchidectomy or leuprolide injections. Orchidectomy induced immediate testosterone deficiency while leuprolide induced testosterone reduction after eight weeks. Due to this difference, orchidectomy delivered more severe patho-physiological complications than leuprolide at 16 weeks. When leuprolide is given, there is a period of testosterone flare which, in humans, lasts for around 1–2 weeks [37]. This is probably the reason for the slower decrease in testosterone concentrations in the leuprolide-treated groups than in the orchidectomized groups. It also means that the exposure to testosterone deficiency in leuprolide-treated rats is shorter than in the orchidectomized groups. Further, orchidectomy induced cardiac inflammation and fibrosis with liver steatosis in the corn starch diet-fed rats whereas leuprolide failed to induced this. It would have been ideal to induce testosterone deficiency at the beginning of the diet initiation, but that would have disrupted the age matching between the groups. Further, we tested Omacor intervention as the reversal protocol in the study and the testosterone deficiency was technically induced in both types of inductions by the start of the Omacor intervention.

Orchidectomy reduced lean mass while increasing fat mass to a greater extent than leuprolide, possibly due to the slower onset of testosterone deficiency with leuprolide. Both orchidectomy and leuprolide increased the deposition of retroperitoneal fat, a major component of the abdominal fat. Presence of abdominal obesity is the major metabolic complication in the initiation of inflammation, potentially increasing the risk of cardiovascular disease [38]. During the protocol, rats were provided with similar environment including cage size and environmental enrichment with no facility to increase physical activity. We have previously shown that exercise in our high-carbohydrate, high-fat diet-fed rats improved metabolic and cardiovascular function [39]. This indicates that the basal physical activity in this study is unlikely to change metabolic or cardiovascular function by itself.

One of the major complications of leuprolide treatment is the development of metabolic syndrome, characterized by the presence of central obesity, hypertension, and dyslipidemia as risk factors for type 2 diabetes and cardiovascular disease [3,4,5,6]. Obesity as the major component of metabolic syndrome is still without an effective, non-invasive treatment free from adverse effects, with functional foods and nutraceuticals, including omega-3 fatty acids, proposed as effective treatment options [40]. EPA and DHA have been reviewed extensively for their effectiveness against metabolic syndrome [13,14,15]. Omega-3 and omega-6 fatty acids play vital roles in regulating metabolism as well as state of inflammation [41,42]. A balance is required between pro-inflammatory omega-6 and anti-inflammatory omega-3 fatty acids for the body to maintain homeostasis [42]. With the increases in omega-6 fatty acid intake in the modern diet, supplementation of omega-3 fatty acids to this diet will reduce the imbalance between omega-3 and omega-6 intake and hence contribute in alleviating metabolic and inflammation-related complications [42]. For omega-3 fatty acids, animal studies described anti-obesity effects, but human studies do not conclusively suggest this [43]. Further, conflicting results are available from the randomized trials of omega-3 fatty acids which may have resulted from differences in study design, dosage used, omega-6/omega-3 fatty acid ratio of the background diet, duration of omega-3 fatty acid supplementation, use of other supplements in addition to omega-3 fatty acids, and demographics of the study population [42].

This study used a commercial mixture of the ethyl esters of EPA and DHA, Omacor, to identify the beneficial effects in the treatment of leuprolide-treated obese rats. Omacor reduced body weight gain during the intervention period without changing body weight. Further, Omacor increased whole-body fat mass and abdominal fat in testosterone-deficient obese rats while decreasing these in testosterone-deficient lean rats. This difference could be due to the differences in the fat content of the two diets. H diet contains higher fat content whereas the C diet is extremely low in fat content [21]. We have previously shown that the proportion of fatty acids in the dietary lipid pool determines the responses to omega-3 fatty acids [44]. Pure EPA and DHA individually showed similar responses to diet-induced metabolic syndrome in C and H rats where low-fat diet-fed rats showed greater responses to omega-3 fatty acids than high-fat diet-fed rats [44]. Further, Omacor treatment improved bone mineral density, plasma concentrations of total cholesterol, triglycerides, and NEFA, and basal blood glucose concentrations. Omega-3 fatty acids attenuated obesity and glucose intolerance and decreased plasma triglycerides in humans [45,46,47]. Further, EPA and DHA suppressed the production of pro-inflammatory cytokines including IL-6, TNF-α, IL-1, and IL-2 [48]. Omega-3 fatty acids upregulated lipoprotein lipase and adipose triglyceride lipase, enzymes catalyzing hydrolysis of triglycerides in skeletal muscle and adipose tissue, respectively [49]. Further, omega-3 fatty acids played crucial roles in lowering the rate of fatty acid synthesis and glucose metabolism through downregulation of fatty acid synthase in liver [50]. Our study results also suggest that omega-3 fatty acid supplementation improved cardiovascular responses including reduced systolic blood pressure and left ventricular diastolic stiffness along with reduced inflammatory cell infiltration and collagen deposition. Further, Omacor reduced lipid accumulation and inflammatory cell infiltration without changing wet weight of liver and its enzyme activities. Omega-3 fatty acids improved endoplasmic reticulum and mitochondrial function in patients with non-alcoholic steatohepatitis [51]. Modulation of nuclear transcription factor activities, such as peroxisome proliferator-activated receptors, sterol regulatory element-binding protein 1c, and carbohydrate-responsive element-binding protein, have been suggested as some of the mechanisms in improving liver lipid metabolism [52,53].

The mechanism of action of omega-3 fatty acids is based on their anti-inflammatory responses [18]. Omega-3 fatty acids protected against metabolic syndrome through their anti-inflammatory and platelet activating properties that enhance endothelial function and normalize blood pressure by restricting the lipogenesis and activation of lipid oxidation [54]. In our previous study, EPA and DHA individually improved metabolic syndrome in obese rats at the same dose as in this study (3% in food) [44]. Colon-specific delivery of EPA or DHA increased the release of glucagon-like peptide 1 and insulin with subsequent reduction in glucose concentrations [55]. Increasing adiponectin is one mechanism by which omega-3 fatty acids can improve cardiometabolic profile in people with cardiovascular risk [56]. Plasma adiponectin was associated with insulin sensitivity [57] and reduced plasma adiponectin was a marker of insulin resistance and increased risk of type 2 diabetes [58]. Both animal and human studies showed that omega-3 fatty acid supplements improved plasma adiponectin concentrations [56,59,60,61]. Thus, increasing both glucagon-like peptide 1 secretion and adiponectin production by omega-3 fatty acids could improve both insulin secretion and sensitivity [62] resulting in improved recovery from insulin resistance and dyslipidemia, thus attenuating metabolic syndrome. Measuring glucagon-like peptide 1 and adiponectin before and after intervention with omega-3 fatty acids could support a plausible mechanism of action of EPA and DHA. However, we were unable to define these hormonal changes making it a limitation of our study. Recently, omega-3 fatty acids have been linked with the browning of white adipose tissue, which would result in the burning of excess fat and loss of heat generated through this non-shivering thermogenesis [42]. Browning of cardiac and vascular adipose tissue has been proposed to reduce cardiovascular disease [63]. Omega-3 fatty acids have been involved in regulation of mitochondrial and endoplasmic reticulum stress that are contributing factors in obesity and insulin resistance [64]. Thus, identifying effects of Omacor on diet-induced obesity through these mechanisms such as increased brown adipose tissue or browning of white adipose tissue warrants future investigation.

## 4. Materials and Methods

### 4.1. Rats and Diets

All experimental procedures were approved by the Animal Ethics Committee of the University of Southern Queensland under the guidelines of the National Health and Medical Research Council of Australia (approval number 11REA004, June 2011). Eight- to nine-week-old male Wistar rats (338 ± 1 g, *n* = 96) were obtained from the Animal Resource Centre (Murdoch, WA, Australia). Rats were randomly divided into 8 groups (Figure 1) each of 12 rats, with 4 groups fed a corn starch diet and 4 groups fed a high-carbohydrate, high-fat diet, as follows:

- **C**: Corn starch diet-fed rats for 16 weeks
- **COr**: Orchidectomized rats fed corn starch diet for 16 weeks
- **CL**: Rats treated with leuprolide and fed corn starch diet for 16 weeks
- **CLOm**: Rats treated with leuprolide for 16 weeks and fed corn starch diet for first 8 weeks followed by corn starch diet supplemented with 3% Omacor for the final 8 weeks
- **H**: High-carbohydrate, high-fat diet-fed rats for 16 weeks
- **HOr**: Orchidectomized rats fed high-carbohydrate, high-fat diet for 16 weeks
- **HL**: Rats treated with leuprolide and fed high-carbohydrate, high-fat diet for 16 weeks
- **HLOm**: Rats treated with leuprolide for 16 weeks and fed high-carbohydrate, high-fat diet for first 8 weeks followed by high-carbohydrate, high-fat diet supplemented with 3% Omacor for the final 8 weeks

### 4.2. Induction of Testosterone Deficiency

Twenty-four male rats were orchidectomized at the age of 8 weeks. Bilateral orchidectomy was performed under anesthesia induced by intraperitoneal injection of Zoletil (tiletamine 15 mg/kg, zolazepam 15 mg/kg; Virbac, Peakhurst, NSW, Australia) combined with Rompun (xylazine 10 mg/kg; Troy Laboratories, Smithfield, NSW, Australia). An incision was made at the midpoint of the scrotum and the underlying tissue, followed by excision of the testicles and part of the spermatic cord. The incision site was sutured and rats were allowed to recover with administration of carprofen (1 mg/kg for 3 days). Before initiation of the experimental diet, the orchidectomized rats were given standard laboratory chow diet and monitored daily. In a further 24 age-matched male rats, testosterone deficiency was induced by subcutaneous injection of 2 mg/kg leuprolide as the acetate (Lupron Depot, AbbVie, Sydney, NSW, Australia) at 0, 4, 8, and 12 weeks of the protocol.

### 4.3. Rats, Diets, and Treatments

All rats were individually housed at the University of Southern Queensland animal house under temperature-controlled, 12-h-light/dark conditions and were fed ad libitum with their respective diets. C, COr, and CL rats were fed with corn starch diet for 16 weeks while CLOm rats were fed with corn starch diet for first 8 weeks and then 3% Omacor-supplemented corn starch diet for the last 8 weeks. C, COr, CL, and CLOm rats received normal drinking water for the duration of the protocol. H, HOr, and HL rats were fed with high-carbohydrate, high-fat diet for 16 weeks while HLOm rats were fed with high-carbohydrate, high-fat diet for first 8 weeks and then 3% Omacor-supplemented high-carbohydrate, high-fat diet for the last 8 weeks. H, HOr, HL, and HLOm rats received drinking water with 25% fructose (*w*/*v*) for the duration of the protocol. Corn starch diet contained 570 g corn starch, 155 g powdered rat food, 25 g Hubble, Mendel and Wakeman salt mixture, and 250 mL water per kilogram of diet. High-carbohydrate, high-fat diet contained 175 g fructose, 395 g sweetened condensed milk, 200 g beef tallow, 155 g powdered rat food, 25 g Hubble, Mendel and Wakeman salt mixture, and 50 mL water per kilogram of diet [21]. These diets were mixed in the laboratory using the ingredients bought from the local supermarket (condensed milk), butchery (beef tallow), or commercial suppliers (powdered rat food, fructose, and salt mixture). The energy densities of the C and H diet were 11.23 kJ/g and 17.83 kJ/g of food, respectively and an additional 3.85 kJ/mL in the drinking water for the H, HOr, HL and HOm rats. In C diet, carbohydrates, proteins, and fats provided 92%, 5%, and 3% energy while in H diet, carbohydrates, proteins, and fats provided 47%, 5%, and 48% energy. Each gram of Omacor contained 840 mg of the omega-3 fatty acid ethyl esters comprising 460 mg of EPA ethyl ester and 380 mg of DHA ethyl ester.

### 4.4. Physiological Parameters

Body weight, and food and water intakes of all rats were measured daily [21]. Abdominal circumference was measured using a standard measuring tape under light sedation with Zoletil (tiletamine 10 mg/kg, zolazepam 10 mg/kg, intraperitoneal). Feed efficiency was calculated as (mean body weight gain (in grams)/daily energy intake (in kJ)) [21].

### 4.5. Systolic Blood Pressure Measurements

Systolic blood pressure was determined every fourth week under light sedation with Zoletil (tiletamine 10 mg/kg, zolazepam 10 mg/kg, intraperitoneal), using an MLT1010 Piezo-Electric Pulse Transducer and inflatable tail-cuff connected to an MLT844 Physiological Pressure Transducer and PowerLab data acquisition unit [21]. After blood pressure measurements, a small volume of blood was collected from the tail vein for measuring plasma total testosterone concentrations.

### 4.6. Echocardiography

Echocardiographic examinations (Phillips iE33, 12-MHz transducer, New York NY, USA) were performed in rats at the end of protocol [21]. Briefly, rats were anesthetized using Zoletil (tiletamine 10 mg/kg and zolazepam 10 mg/kg, intraperitoneal) and Ilium Xylazil (xylazine 6 mg/kg, intraperitoneal) and positioned in dorsal recumbency before scanning [21].

### 4.7. Body Composition Measurement

Dual-energy X-ray absorptiometric (DXA) measurements were carried out at the end of the protocol with a Norland XR36 DXA instrument (Norland Corp, Fort Atkinson, WI, USA). These scans were evaluated using the manufacturer’s suggested software for use in laboratory animals (Small Subject Analysis Software, version 2.5.3/1.3.1; Norland Corp) [65]. The precision error of lean mass for replicate measurements, with repositioning, was 3.2%.

### 4.8. Oral Glucose Tolerance Test

Oral glucose tolerance tests were performed on rats every fourth week following a 12-h food deprivation when fructose-supplemented drinking water in all H diet-fed groups was replaced with normal drinking water [21]. After determining basal blood glucose concentrations in tail vein blood using Medisense Precision Q.I.D. glucose meters (Abbott Laboratories, Bedford, MA, USA ), rats were given a glucose load of 2 g/kg body weight as 40% glucose solution via oral gavage and blood glucose concentrations were measured again 30, 60, 90, and 120 min after oral glucose administration [21].

### 4.9. Terminal Experiments

Rats were euthanized with Lethabarb (pentobarbitone sodium, 100 mg/kg, intraperitoneal) before injection of heparin (200 IU) through the right femoral vein. The abdomen was then opened and blood (~5 mL) was withdrawn from the abdominal aorta and collected into heparinized tubes. Blood was centrifuged at 5000× *g* for 15 min to obtain plasma. Plasma was stored at −20 °C for further biochemical characterization. Hearts were then removed for isolated Langendorff heart studies.

### 4.10. Left Ventricular Function

Isolated Langendorff heart preparations were used to assess left ventricular function of rats [21]. Hearts isolated from euthanized rats were perfused with modified Krebs-Henseleit bicarbonate buffer bubbled with 95% O_2_/5% CO_2_ and maintained at 35 °C. Isovolumetric ventricular function was measured by inserting a latex balloon catheter into the left ventricle connected to a Capto SP844 MLT844 physiological pressure transducer (ADInstruments, Sydney, NSW, Australia) and Chart software (5.0, ADInstruments, Sydney, NSW, Australia) on a Maclab system. Left ventricular end-diastolic pressure values were measured during pacing of the heart at 250 beats per minute using an electrical stimulator. End-diastolic pressures were obtained from 0 to 30 mmHg for the calculation of diastolic stiffness constant (κ, dimensionless) [21].

### 4.11. Vascular Reactivity

Thoracic aortic rings (~4 mm in length) were suspended in an organ bath filled with Tyrode physiological salt solution bubbled with 95% O_2_–5% CO_2_ maintained at 35 °C and the rings were allowed to stabilize at a resting tension of ~10 mN. Cumulative concentration–response curves (contraction) were obtained for noradrenaline and cumulative concentration–response curves (relaxation) were obtained for acetylcholine and sodium nitroprusside after submaximal (~70%) contraction to noradrenaline [21].

### 4.12. Organ Weights

After isolated heart perfusion studies, hearts were separated into left ventricles (with septum) and right ventricles and weighed. Livers were isolated and weighed. Retroperitoneal and omental fat pads were removed separately and weighed; epididymal fat pads were removed from rats except orchidectomized rats. Organ weights were normalized against the tibial length at the time of organ removal and expressed as mg/mm of tibial length [21].

### 4.13. Histology

Heart and liver portions were collected and fixed in 10% neutral buffered formalin. The samples were then dehydrated and embedded in paraffin wax. Thin sections (~5 µm) of heart and liver were cut and stained with hematoxylin and eosin to study infiltration of inflammatory cells and for determining fat vacuoles in liver. Heart sections were also stained with picrosirius red stain to study collagen distribution in the heart [21].

### 4.14. Plasma Biochemistry

Activities of aspartate transaminase (AST) and alanine transaminase (ALT), and concentrations of total cholesterol, triglycerides, and non-esterified fatty acids (NEFA) in plasma were measured [21]. Plasma total testosterone concentrations were measured using commercial kits (Enzo Life Sciences, Farmingdale, NY, USA) according to protocols provided by the manufacturer.

### 4.15. Statistical Analysis

All data are presented as mean ± SEM. Results were tested for variance using Bartlett’s test and variables that were not normally distributed were transformed (using log 10 function) prior to statistical analyses. C, COr, HL, H, HL, and HOr rats were tested for effects of diet, testosterone deficiency and their interactions by two-way analysis of variance. When the interaction and/or the main effects were significant, means were compared using Newman–Keuls multiple comparison post hoc test. CL, CLOm, HL, and HLOm groups were tested for effects of diet + leuprolide, Omacor treatment and their interactions by two-way analysis of variance. When the interaction and/or the main effects were significant, means were compared using Newman–Keuls multiple comparison post hoc test. *p* value of <0.05 was considered significant. All statistical analyses were performed using GraphPad Prism version 6.1 for Windows (San Diego, CA, USA).

## 5. Conclusions

We demonstrated that orchidectomy and treatment with a GnRH agonist produced similar worsening of metabolic syndrome symptoms and cardiovascular function. Omacor, a combination of ethyl esters of EPA and DHA, delivered positive physiological and biochemical responses to reduce symptoms of metabolic syndrome. Further, this study is consistent with previous outcomes obtained for omega-3 fatty acids except for attenuating visceral obesity. Reduced systolic blood pressure and left ventricular stiffness were the major cardiovascular outcomes from this study. Given these observations and the ease of administration, clinical trials of Omacor in men with prostate cancer being managed with androgen deprivation therapy are warranted.

## Figures and Tables

**Table 1:** Effects of orchidectomy and leuprolide-induced changes in physiological and metabolic parameters.

Variables	C	COr	CL	H	HOr	HL	*p* Value		
							Diet	Testosterone Deficiency	Diet × Testosterone Deficiency
Initial body weight (g)	337 ± 1	338 ± 1	337 ± 1	336 ± 1	339 ± 1	338 ± 1	0.68	0.14	0.52
Final body weight (g)	419 ± 5 ^b^	429 ± 5 ^b^	417 ± 8 ^b^	520 ± 6 ^a^	522 ± 14 ^a^	539 ± 18 ^a^	<0.0001	0.71	0.37
Body weight gain 9–16 weeks (%)	5.3 ± 0.9 ^c^	11.0 ± 1.2 ^b^	11.2 ± 1.1 ^b^	19.3 ± 0.9 ^a^	17.6 ± 1.6 ^a^	20.4 ± 0.9 ^a^	<0.0001	0.011	0.006
Water intake (mL/day)	31.0 ± 2.1 ^a^	32.0 ± 2.4 ^a^	26.4 ± 2.6 ^a,b^	19.8 ± 1.6 ^b,c^	19.8 ± 1.6 ^b,c^	17.5 ± 1.7 ^c^	<0.0001	0.12	0.71
Food intake (g/day)	31.6 ± 2.0 ^a^	31.9 ± 2.3 ^a^	36.1 ± 2.2 ^a^	20.8 ± 1.3 ^b^	21.1 ± 1.8 ^b^	24.3 ± 1.9 ^b^	<0.0001	0.08	0.96
Energy intake (kJ/day)	346 ± 3 ^d^	356 ± 3 ^d^	401 ± 6 ^c^	444 ± 3 ^b^	447 ± 5 ^b^	507 ± 12 ^a^	<0.0001	<0.0001	0.54
Feed efficiency (g/kJ)	0.23 ± 0.01 ^b^	0.25 ± 0.02 ^b^	0.20 ± 0.02 ^b^	0.41 ± 0.01 ^a^	0.40 ± 0.03 ^a^	0.40 ± 0.03 ^a^	<0.0001	0.48	0.51
Abdominal circumference (cm)	18.4 ± 0.1 ^d^	20.1 ± 0.4 ^c^	20.1 ± 0.2 ^c^	22.0 ± 0.2 ^b^	23.2 ± 0.5 ^a^	23.0 ± 0.3 ^a^	<0.0001	<0.0001	0.52
Basal blood glucose (mmol/L)	4.1 ± 0.1 ^d^	4.7 ± 0.1 ^c^	4.8 ± 0.1 ^c^	5.1 ± 0.1 ^b,c^	5.6 ± 0.1 ^a^	5.4 ± 0.2 ^a,b^	<0.0001	<0.0001	0.24
Area under the curve (mmol/L × minutes)	632 ± 21 ^c^	776 ± 19 ^b^	781 ± 10 ^b^	774 ± 16 ^b^	854 ± 14 ^a^	844 ± 24 ^a^	<0.0001	<0.0001	0.07
Total cholesterol (mmol/L)	1.37 ± 0.05 ^b^	1.71 ± 0.15 ^a,b^	1.65 ± 0.04 ^a,b^	1.88 ± 0.06 ^a^	1.84 ± 0.17 ^a^	1.90 ± 0.10 ^a^	0.001	0.28	0.20
Triglyceride (mmol/L)	0.60 ± 0.03 ^c^	0.56 ± 0.05 ^c^	0.60 ± 0.06 ^c^	1.42 ± 0.15 ^b^	1.06 ± 0.22 ^b,c^	2.02 ± 0.32 ^a^	<0.0001	0.019	0.032
NEFA (mmol/L)	1.74 ± 0.16 ^c^	2.47 ± 0.17 ^c^	2.38 ± 0.19 ^c^	3.69 ± 0.37 ^b^	3.41 ± 0.43 ^b^	4.60 ± 0.35 ^a^	<0.0001	0.034	0.09
Whole-body lean mass (g)	312 ± 11 ^a^	271 ± 11 ^b^	275 ± 5 ^b^	314 ± 8 ^a^	229 ± 14 ^c^	308 ± 9 ^a^	0.78	<0.0001	0.002
Whole-body fat mass (g)	114 ± 11 ^c^	121 ± 10 ^c^	112 ± 8 ^c^	191 ± 8 ^b^	259 ± 23 ^a^	161 ± 15 ^b,c^	<0.0001	0.0007	0.006
Bone mineral content (g)	13.3 ± 0.5 ^b^	12.4 ± 0.4 ^b,c^	11.9 ± 0.2 ^b,c^	13.3 ± 0.4 ^b^	16.2 ± 0.6 ^a^	11.3 ± 0.5 ^c^	0.005	<0.0001	<0.0001
Bone mineral density (g/cm^2^)	0.183 ± 0.003	0.173 ± 0.004	0.174 ± 0.003	0.183 ± 0.004	0.173 ± 0.003	0.177 ± 0.003	0.72	0.12	0.88
Retroperitoneal fat (mg/mm)	122 ± 8 ^d^	231 ± 18 ^c^	220 ± 18 ^c^	366 ± 17 ^b^	537 ± 26 ^a^	479 ± 54 ^a^	<0.0001	<0.0001	0.51
Epididymal fat (mg/mm)	101 ± 4 ^b^	-	125 ± 11 ^b^	224 ± 17 ^a^	-	263 ± 26 ^a^	<0.0001	0.07	0.65
Omental fat (mg/mm)	65 ± 6 ^b^	90 ± 6 ^b^	83 ± 6 ^b^	132 ± 9 ^a^	137 ± 13 ^a^	147 ± 13 ^a^	<0.0001	0.16	0.52

Values are means ± SEM, *n* = 10–12. Mean values in a row with unlike superscript letters (a–d) are significantly different (*p* < 0.05). C, corn starch diet-fed rats; COr, orchidectomized rats fed corn starch diet; CL, leuprolide-treated rats fed corn starch diet; H, high-carbohydrate high-fat diet-fed rats; HOr, orchidectomized rats fed high-carbohydrate high-fat diet; HL, leuprolide-treated rats fed high-carbohydrate high-fat diet; NEFA, non-esterified fatty acids.

**Table 2:** Effects of orchidectomy and leuprolide-induced changes in cardiovascular and liver function.

Variables	C	COr	CL	H	HOr	HL	*p* Value		
							Diet	Testosterone Deficiency	Diet × Testosterone Deficiency
Heart rate (bpm)	253 ± 23 ^b,c^	227 ± 17 ^c^	238 ± 11 ^b,c^	350 ± 10 ^a^	282 ± 15 ^b,c^	290 ± 15 ^b^	<0.0001	0.01	0.29
LVIDd (mm)	6.64 ± 0.28 ^c^	6.63 ± 0.30 ^c^	7.70 ± 0.15 ^a,b^	7.09 ± 0.22 ^b,c^	7.23 ± 0.20 ^b,c^	8.24 ± 0.22 ^a^	0.007	<0.0001	0.95
LVIDs (mm)	3.11 ± 0.19 ^c^	3.68 ± 0.18 ^b,c^	4.52 ± 0.19 ^a^	3.22 ± 0.23 ^c^	3.48 ± 0.25 ^b,c^	4.14 ± 0.18 ^a,b^	0.35	<0.0001	0.49
IVSd (mm)	1.91 ± 0.12	1.96 ± 0.10	1.77 ± 0.06	2.05 ± 0.08	2.05 ± 0.17	1.82 ± 0.06	0.28	0.10	0.91
IVSs (mm)	3.09 ± 0.26 ^a,b^	3.13 ± 0.20 ^a,b^	2.80 ± 0.08 ^b^	3.65 ± 0.09 ^a^	3.43 ± 0.20 ^a,b^	3.12 ± 0.11 ^a,b^	0.006	0.047	0.70
LVPWd (mm)	1.70 ± 0.09	1.91 ± 0.12	1.79 ± 0.03	2.04 ± 0.06	2.08 ± 0.15	1.75 ± 0.05	0.05	0.06	0.14
LVPWs (mm)	2.73 ± 0.16 ^b^	2.65 ± 0.14 ^b^	2.58 ± 0.15 ^b^	3.27 ± 0.14 ^a^	3.04 ± 0.13 ^a,b^	2.95 ± 0.10 ^a,b^	0.0003	0.23	0.80
Diastolic volume (µL)	317 ± 41 ^c^	318 ± 38 ^c^	481 ± 26 ^a,b^	427 ± 40 ^a,b,c^	356 ± 30 ^b,c^	531 ± 47 ^a^	0.036	0.0001	0.59
Systolic volume (µL)	34.2 ± 6.1 ^c^	55.0 ± 8.2 ^b,c^	100.2 ± 12.8 ^a^	38.4 ± 7.0 ^c^	49.2 ± 10.5 ^b,c^	77.1 ± 8.6 ^a,b^	0.28	<0.0001	0.33
Stroke volume (µL)	283 ± 38 ^b^	263 ± 33 ^b^	381 ± 22 ^a,b^	388 ± 35 ^a,b^	306 ± 24 ^b^	454 ± 43 ^a^	0.009	0.0007	0.65
SBP:LVIDs	42.4 ± 2.9 ^a,b^	40.9 ± 1.8 ^a,b,c^	32.1 ± 1.5 ^c^	48.3 ± 3.8 ^a^	48.0 ± 3.0 ^a^	36.9 ± 2.4 ^b,c^	0.009	0.0001	0.91
SBP:systolic volume	4826 ± 956 ^a,b^	3016 ± 418 ^a,b^	1599 ± 198 ^b^	5562 ± 1462 ^a^	4436 ± 840 ^a,b^	2291 ± 479 ^a,b^	0.17	0.001	0.89
ESS:LVIDs	2.40 ± 0.10 ^b^	2.44 ± 0.08 ^a,b^	2.84 ± 0.16 ^a^	2.30 ± 0.09 ^b^	2.69 ± 0.09 ^a,b^	2.56 ± 0.11 ^a,b^	0.63	0.007	0.05
Cardiac output (mL/min)	70.1 ± 9.2 ^b^	56.3 ± 7.9 ^b^	90.6 ± 7.3 ^b^	135.2 ± 10.9 ^a^	84.8 ± 5.6 ^b^	130.9 ± 12.9 ^a^	<0.0001	0.0001	0.14
Relative wall thickness	0.56 ± 0.05	0.58 ± 0.05	0.47 ± 0.02	0.56 ± 0.02	0.60 ± 0.05	0.45 ± 0.02	1.00	0.003	0.87
Systolic wall stress	75.7 ± 7.0 ^b^	104.4 ± 8.9 ^a,b^	129.9 ± 12.2 ^a^	74.8 ± 7.1 ^b^	93.3 ± 6.6 ^b^	106.4 ± 7.3 ^a,b^	0.09	<0.0001	0.41
Estimated LV mass (g)	0.81 ± 0.03 ^b^	0.89 ± 0.05 ^a,b^	1.01 ± 0.04 ^a,b^	1.16 ± 0.07 ^a^	1.08 ± 0.12 ^a,b^	1.07 ± 0.06 ^a,b^	0.006	0.65	0.11
Fractional shortening (%)	53.1 ± 2.3 ^a,b^	47.1 ± 2.1 ^b,c^	41.2 ± 2.1 ^c^	56.6 ± 2.5 ^a^	49.6 ± 2.7 ^a,b,c^	47.7 ± 2.3 ^b,c^	0.033	0.0002	0.68
Ejection fraction (%)	89.1 ± 1.6 ^a^	84.0 ± 2.0 ^a,b^	79.3 ± 2.0 ^b^	91.2 ± 1.5 ^a^	86.6 ± 2.1 ^a^	85.2 ± 1.7 ^a,b^	0.021	0.0002	0.53
Systolic blood pressure (mmHg)	126 ± 2 ^d^	146 ± 1 ^b,c^	144 ± 2 ^c^	152 ± 2 ^b^	161 ± 2 ^a^	150 ± 2 ^b,c^	<0.0001	<0.0001	<0.0001
Right ventricular wet weight (mg/mm)	2.37 ± 0.19	3.39 ± 0.64	2.76 ± 0.16	4.21 ± 1.14	2.88 ± 0.12	3.19 ± 0.15	0.20	0.85	0.11
Left ventricular + septum wet weight (mg/mm)	17.6 ± 0.5 ^b^	18.1 ± 0.7 ^a,b^	18.1 ± 0.5 ^a,b^	19.5 ± 0.5 ^a,b^	19.9 ± 0.2 ^a^	19.1 ± 0.5 ^a,b^	0.003	0.62	0.62
Left ventricular diastolic stiffness constant	23.8 ± 0.7 ^b^	29.4 ± 0.6 ^a^	27.3 ± 0.5 ^a^	29.1 ± 0.7 ^a^	29.1 ± 1.1 ^a^	27.9 ± 0.7 ^a^	0.003	0.002	0.0006
Liver wet weight (mg/mm)	241 ± 7 ^b^	239 ± 7 ^b^	250 ± 15 ^b^	333 ± 8 ^a^	351 ± 11 ^a^	325 ± 19 ^a^	<0.0001	0.76	0.31
ALT activity (U/L)	25.9 ± 1.6 ^b^	29.0 ± 2.6 ^b^	27.6 ± 1.9 ^b^	41.8 ± 2.0 ^a^	39.6 ± 1.6 ^a^	40.4 ± 5.9 ^a^	<0.0001	0.99	0.68
AST activity (U/L)	64.3 ± 2.9 ^b^	78.3 ± 5.5 ^a,b^	64.1 ± 3.5 ^b^	88.3 ± 3.6 ^a^	95.1 ± 4.3 ^a^	86.7 ± 9.4 ^a^	<0.0001	0.07	0.78

Values are means ± SEM, *n* = 10–12. Mean values in a row with unlike superscript letters (a–d) are significantly different (*p* < 0.05). C, corn starch diet-fed rats; COr, orchidectomized rats fed corn starch diet; CL, leuprolide-treated rats fed corn starch diet; H, high-carbohydrate high-fat diet-fed rats; HOr, orchidectomized rats fed high-carbohydrate high-fat diet; HL, leuprolide-treated rats fed high-carbohydrate high-fat diet; LVIDd, left ventricular internal diameter during diastole; LVIDs, left ventricular internal diameter during systole; IVSd, interventricular septal thickness during diastole; IVSs, interventricular septal thickness during systole; LVPWd, left ventricular posterior wall thickness during diastole; LVPWs. left ventricular posterior wall thickness during systole; ESS, end-systolic stress; AST, aspartate transaminase; ALT, alanine transaminase.

**Table 3:** Effects of Omacor on leuprolide-induced changes in physiological and metabolic parameters.

Variables	CL	CLOm	HL	HLOm	*p* Value		
					Diet + Leuprolide	Omacor	(Diet + Leuprolide) × Omacor
Initial body weight (g)	337 ± 1	342 ± 2	338 ± 1	339 ± 3	0.61	0.13	0.31
Final body weight (g)	417 ± 8 ^b^	319 ± 9 ^c^	539 ± 18 ^a^	536 ± 19 ^a^	<0.0001	0.001	0.002
Body weight gain 9–16 weeks (%)	11.2 ± 1.1 ^c^	−14.0 ± 1.4 ^d^	20.4 ± 0.9 ^a^	15.3 ± 1.5 ^b^	<0.0001	<0.0001	<0.0001
Water intake (mL/day)	26.4 ± 2.6 ^b^	33.2 ± 2.6 ^a^	17.5 ± 1.7 ^c^	24.5 ± 1.1 ^b^	0.0001	0.002	0.96
Food intake (g/day)	36.1 ± 2.2 ^a^	38.3 ± 2.8 ^a^	24.3 ± 1.9 ^b^	26.1 ± 1.1 ^b^	<0.0001	0.34	0.93
Energy intake (kJ/day)	401 ± 6 ^c^	487 ± 34 ^b^	507 ± 12 ^a,b^	560 ± 21 ^a^	0.0001	0.002	0.44
Feed efficiency (g/kJ)	0.20 ± 0.02 ^b^	−0.03 ± 0.01 ^c^	0.40 ± 0.03 ^a^	0.35 ± 0.03 ^a^	<0.0001	<0.0001	0.0005
Abdominal circumference (cm)	20.1 ± 0.2 ^b^	17.0 ± 0.2 ^c^	23.0 ± 0.4 ^a^	22.0 ± 0.4 ^a^	<0.0001	<0.0001	0.002
Basal blood glucose (mmol/L)	4.8 ± 0.1 ^b^	3.5 ± 0.1 ^c^	5.4 ± 0.2 ^a^	4.5 ± 0.3 ^b^	0.0002	<0.0001	0.31
Area under the curve (mmol/L × minutes)	781 ± 10 ^b^	599 ± 16 ^c^	844 ± 24 ^b^	933 ± 40 ^a^	<0.0001	0.07	<0.0001
Total cholesterol (mmol/L)	1.65 ± 0.05 ^b^	1.04 ± 0.03 ^d^	1.89 ± 0.11 ^a^	1.41 ± 0.07 ^c^	0.0001	<0.0001	0.37
Triglyceride (mmol/L)	0.60 ± 0.06 ^b^	0.27 ± 0.02 ^b^	1.98 ± 0.31 ^a^	0.33 ± 0.04 ^b^	<0.0001	<0.0001	0.0004
NEFA (mmol/L)	2.38 ± 0.19 ^b^	0.46 ± 0.03 ^c^	4.63 ± 0.43 ^a^	0.99 ± 0.18 ^c^	<0.0001	<0.0001	0.0016
Whole-body lean mass (g)	314 ± 8 ^a^	261 ± 10 ^b^	308 ± 9 ^a^	251 ± 13 ^b^	0.44	<0.0001	0.85
Whole-body fat mass (g)	191 ± 8 ^b^	59 ± 8 ^c^	161 ± 15 ^b^	248 ± 27 ^a^	<0.0001	0.18	<0.0001
Bone mineral content (g)	0.173 ± 0.003	0.179 ± 0.003	0.177 ± 0.004	0.180 ± 0.001	0.40	0.14	0.62
Bone mineral density (g/cm^2^)	12.0 ± 0.2 ^b^	10.6 ± 0.5 ^b^	11.3 ± 0.5 ^b^	16.8 ± 0.9 ^a^	<0.0001	0.001	<0.0001
Retroperitoneal fat (mg/mm)	220 ± 18 ^b^	95 ± 14 ^c^	479 ± 54 ^a^	406 ± 50 ^a^	<0.0001	0.015	0.50
Epididymal fat (mg/mm)	125 ± 11 ^b^	67 ± 7 ^c^	263 ± 26 ^a^	226 ± 26 ^a^	<0.0001	0.02	0.59
Omental fat (mg/mm)	83 ± 6 ^c^	79 ± 10 ^c^	147 ± 13 ^b^	249 ± 20 ^a^	<0.0001	0.0007	0.0003

Values are means ± SEM, *n* = 10–12. Mean values in a row with unlike superscript letters (a–d) are significantly different (*p* < 0.05). CL, leuprolide-treated rats fed corn starch diet; CLOm, leuprolide-treated rats fed corn starch diet supplemented with Omacor; HL, leuprolide-treated rats fed high-carbohydrate high-fat diet; HLOm, leuprolide-treated rats fed high-carbohydrate, high-fat diet supplemented with Omacor; NEFA, non-esterified fatty acids.

**Table 4:** Effects of Omacor on leuprolide-induced changes in cardiovascular and liver function.

Variables	CL	CLOm	HL	HLOm	*p* Value		
					Diet + Leuprolide	Omacor	(Diet + Leuprolide) × Omacor
Heart rate	238 ± 11	255 ± 22	290 ± 15	283 ± 11	0.08	0.75	0.44
LVIDd (mm)	7.70 ± 0.15 ^b^	7.64 ± 0.17 ^b^	8.24 ± 0.22 ^a,b^	8.54 ± 0.16 ^a^	0.0002	0.50	0.32
LVIDs (mm)	4.52 ± 0.19 ^b^	4.62 ± 0.13 ^b^	4.14 ± 0.18 ^b^	5.33 ± 0.09 ^a^	0.29	0.0001	0.0009
IVSd (mm)	1.77 ± 0.06 ^a,b^	1.68 ± 0.05 ^b^	1.82 ± 0.06 ^a,b^	1.91 ± 0.02 ^a^	0.008	1.0000	0.08
IVSs (mm)	2.80 ± 0.08 ^b^	2.72 ± 0.07 ^b^	3.12 ± 0.11 ^a^	2.92 ± 0.06 ^a,b^	0.0028	0.10	0.47
LVPWd (mm)	1.79 ± 0.03 ^a,b^	1.67 ± 0.03 ^b^	1.75 ± 0.05 ^a,b^	1.84 ± 0.04 ^a^	0.10	0.70	0.009
LVPWs (mm)	2.58 ± 0.15 ^b^	2.39 ± 0.05 ^b^	2.95 ± 0.10 ^a^	2.65 ± 0.06 ^b^	0.003	0.017	0.58
Diastolic volume (µL)	481 ± 26 ^b^	473 ± 34 ^b^	531 ± 47 ^b^	658 ± 36 ^a^	0.002	0.11	0.07
Systolic volume (µL)	100.0 ± 12.8 ^b^	104.9 ± 8.9 ^b^	77.0 ± 8.6 ^b^	159.2 ± 7.8 ^a^	0.12	<0.0001	0.0003
Stroke volume (µL)	381 ± 22 ^b^	368 ± 25 ^b^	454 ± 43 ^a,b^	499 ± 31 ^a^	0.002	0.61	0.36
SBP:LVIDs	32.1 ± 1.5 ^b^	26.5 ± 0.9 ^c^	36.9 ± 2.4 ^a^	25.9 ± 0.7 ^c^	0.18	<0.0001	0.08
SBP:systolic volume	1599 ± 198 ^a,b^	1216 ± 101 ^b^	2291 ± 479 ^a^	882 ± 48 ^b^	0.50	0.002	0.06
ESS:LVIDs	2.84 ± 0.16	2.55 ± 0.07	2.56 ± 0.11	2.61 ± 0.08	0.33	0.28	0.13
Cardiac output (mL)	91 ± 7 ^b^	95 ± 11 ^b^	131 ± 13 ^a^	141 ± 10 ^a^	0.0001	0.50	0.76
Relative wall thickness	0.47 ± 0.02	0.44 ± 0.01	0.45 ± 0.02	0.44 ± 0.01	0.53	0.21	0.53
Systolic wall stress	129.9 ± 12.2 ^a,b^	117.8 ± 4.0 ^a,b^	106.4 ± 7.3 ^b^	139.0 ± 4.0 ^a^	0.88	0.19	0.006
Estimated LV mass (g)	1.01 ± 0.04 ^b^	0.92 ± 0.05 ^b^	1.07 ± 0.06 ^b^	1.29 ± 0.05 ^a^	0.0001	0.21	0.004
Fractional shortening (%)	41.2 ± 2.1 ^b^	39.6 ± 0.7 ^b^	47.7 ± 2.3 ^a^	37.6 ± 0.8 ^b^	0.18	0.0009	0.013
Ejection fraction (%)	79.3 ± 2.0 ^b^	77.9 ± 0.7 ^b^	85.2 ± 1.7 ^a^	75.6 ± 0.9 ^b^	0.22	0.0004	0.006
Systolic blood pressure (mmHg)	144 ± 2 ^b^	122 ± 1 ^d^	150 ± 2 ^a^	137 ± 2 ^c^	<0.0001	<0.0001	0.016
Right ventricular wet weight (mg/mm)	2.76 ± 0.16 ^c^	3.59 ± 0.29 ^b^	3.19 ± 0.15 ^b,c^	4.71 ± 0.22 ^a^	0.0008	<0.0001	0.11
Left ventricular + septum wet weight (mg/mm)	18.1 ± 0.5 ^b^	17.2 ± 0.8 ^b^	19.1 ± 0.5 ^b^	22.9 ± 0.9 ^a^	<0.0001	0.044	0.002
Left ventricular diastolic stiffness constant	27.3 ± 0.5 ^a^	22.8 ± 1.0 ^bc^	27.9 ± 0.7 ^a^	21.3 ± 1.0 ^c^	0.59	<0.0001	0.21
Liver wet weight (mg/mm)	250 ± 15 ^b^	161 ± 7 ^c^	325 ± 19 ^a^	294 ± 14 ^a^	<0.0001	0.0001	0.05
ALT activity (U/L)	27.6 ± 1.9 ^b^	41.4 ± 4.4 ^a^	34.9 ± 2.4 ^ab^	39.7 ± 2.8 ^a^	0.36	0.004	0.15
AST activity (U/L)	64.1 ± 3.5 ^b^	72.5 ± 3.1 ^ab^	86.7 ± 9.4 ^a^	70.0 ± 4.2 ^ab^	0.08	0.47	0.032

Values are means ± SEM, *n* = 10–12. Mean values in a row with unlike superscript letters (a–d) are significantly different (*p* < 0.05). CL, leuprolide-treated rats fed corn starch diet; CLOm, leuprolide-treated rats fed corn starch diet supplemented with Omacor; HL, leuprolide-treated rats fed high-carbohydrate high-fat diet; HLOm, leuprolide-treated rats fed high-carbohydrate, high-fat diet supplemented with Omacor; LVIDd, left ventricular internal diameter during diastole; LVIDs, left ventricular internal diameter during systole; IVSd, interventricular septal thickness during diastole; IVSs, interventricular septal thickness during systole; LVPWd, left ventricular posterior wall thickness during diastole; LVPWs. left ventricular posterior wall thickness during systole; ESS, end-systolic stress; AST, aspartate transaminase; ALT, alanine transaminase.
